# Supplementary material for: Exogenous Melatonin Alleviates NaCl Injury by Influencing Stomatal Morphology, Photosynthetic Performance, and Antioxidant Balance in Maize
Source: Int J Mol Sci. 2024 Sep 19;25(18):10077. doi: 10.3390/ijms251810077 (PMC11432274; doi:10.3390/ijms251810077)
Supplement: Supplementary file 1 [file ijms-25-10077-s001.zip › Figure S1.pdf]

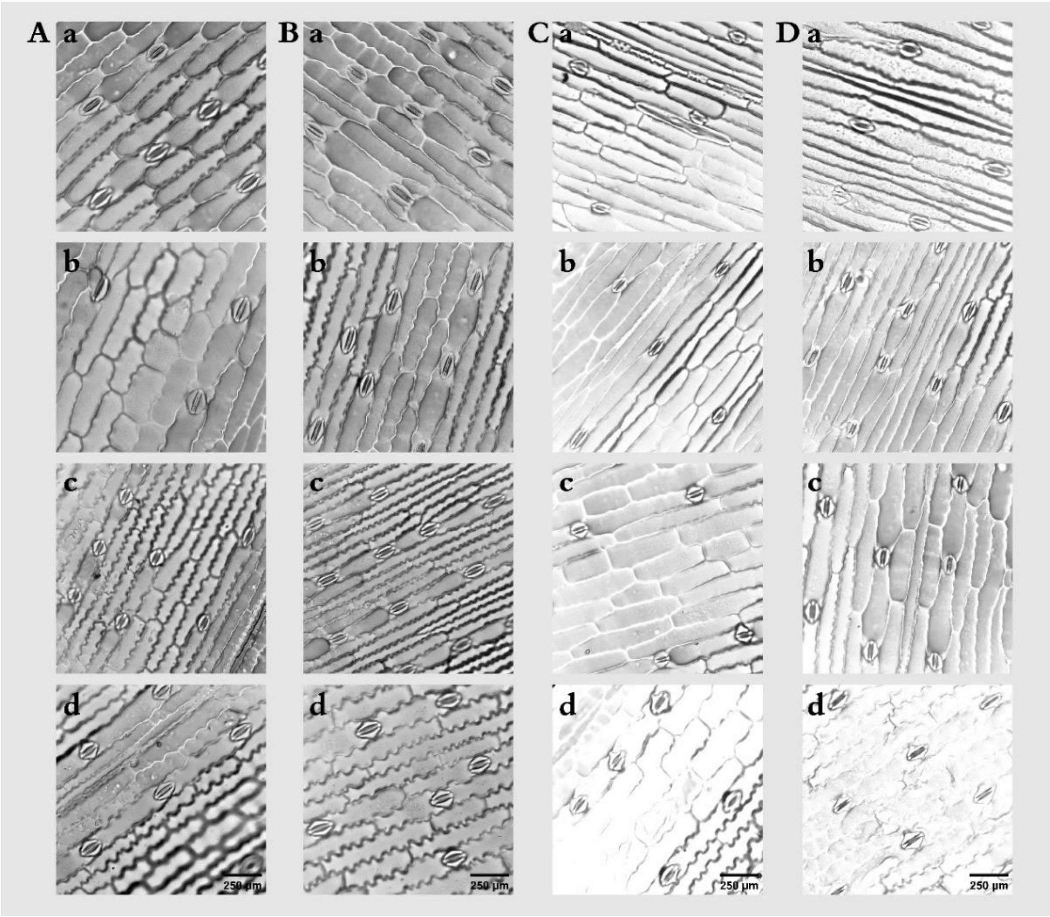

Figure S1. Stomatal morphological characteristics of adaxial stomata (A, C) and abaxial stomata (B, D) of the 3rd leaf in maize seedlings under different treatments. A and C were the adaxial stomata of LX19 and NX40-6 leaves, respectively. B and D were the abaxial stomata of LX19 and NX40–6 leaves. Thea, b, c and d were the four treatments including CK, M–CK, SS, and M–SS respectively. CK: 0 mM NaCl+0 μM melatonin (MT) treatment; SS: 180 mM NaCl+0 μM MT treatment; M–CK: 0 mM NaCl+150 μM MT treatment; M–SS: 180 mM NaCl+150 μM MT treatment.
