# Supplementary material for: Exogenous Melatonin Alleviates NaCl Injury by Influencing Stomatal Morphology, Photosynthetic Performance, and Antioxidant Balance in Maize
Source: Int J Mol Sci. 2024 Sep 19;25(18):10077. doi: 10.3390/ijms251810077 (PMC11432274; doi:10.3390/ijms251810077)
Supplement: Supplementary file 1 [file ijms-25-10077-s001.zip › Table S1.pdf]

Table S1. Sequences of primers used in reverse–transcription quantitative PCR (RT–qPCR) and functional annotation of these candidate genes.

Gene ID (Encoded protein)	Gene position	Primer sequence (5' to 3')	Gene functional annotation
Zm00001d009990 (superoxide dismutase [Mn] 3.4, mitochondrial)	Chromosome 8 (93603841_93617355 bp)	F: TTTTGGAAGAACCTCAGCTAT<br>R: CCCAGACATCAATCCCCAAC	superoxide dismutase activity (GO:0004784); oxidoreductase activity (GO:0016491); removal of superoxide radicals (GO:0019430); superoxide metabolic process (GO:0006801);
Zm00001d047479 (superoxide dismutase [Cu-Zn] 4AP)	Chromosome 9 (131494600_131498241 bp)	F: GGCTGTTGCTGTGCTTGGTA<br>R: CTTGCTCGCAGGATTGTAGTG	superoxide dismutase activity (GO:0004784); oxidoreductase activity (GO:0016491); antioxidant activity (GO:0016209); superoxide metabolic process (GO:0006801);
Zm00001d014848 (catalase 1)	Chromosome 5 (65456774_65461269 bp)	F: CAGGCTGTCGTGAGAAGTGC<br>R: GAGATCCAAATGGTAGGTGTT	catalase activity (GO:0004096); peroxidase activity (GO:0004601); oxidoreductase activity (GO:0016491);
Zm00001d007234 (ascorbate peroxidase 2)	Chromosome 2 (225337099_225339736 bp)	F: TGAGCGACCAGGACATTG<br>R: GAGGGCTTTGTCA CTTGGT	L-ascorbate peroxidase activity (GO:0016688); peroxidase activity (GO:0004601); oxidoreductase activity (GO:0016491); hydrogen peroxide catabolic process (GO:0042744); response to reactive oxygen species (GO:0000302);
Zm00001d011819 (Chlorophyllide a oxygenase chloroplastic)	Chromosome 8 (162062135_162065501 bp)	F: CCATCAAGAAGGCAAGTTCC<br>R: TCTTTCCTCAAGGTCCCGAT	chlorophyllide a oxygenase [overall] activity (GO:0010277); oxidoreductase activity (GO:0016491);
Zm00001d017766 (nine–cis–epoxycarotenoid dioxygenase 8)	Chromosome 5 (206198899_206201211 bp)	F: CCACGCACACCAGAGTTACA<br>R: GCTGGGCGCCTTTCTACTAA	carotene catabolic process (GO:0016121); obsolete oxidation-reduction process (GO:0055114); carotenoid dioxygenase activity (GO:0010436); oxidoreductase activity (GO:0016702)
Zm00001d010159 (actin 1)	Chromosome 8 (102413768_102417536 bp)	F: CGATTGAGCATGGCATTGTCA<br>R: CCCACTAGCGTACAACGAA	nucleotide binding (GO:0000166); ATP binding (GO:0005524); cytoskeleton (GO:0005856);
